# Clinical Course, Prognosis, and Cause of Death in Primary Sjögren's Syndrome

**DOI:** 10.1155/2014/647507

**Published:** 2014-05-20

**Authors:** Ildiko Fanny Horvath, Antonia Szanto, Gabor Papp, Margit Zeher

**Affiliations:** Division of Clinical Immunology, Faculty of Medicine, University of Debrecen, Móricz Zsigmond Street 22, Debrecen 4032, Hungary

## Abstract

The aim of this retrospective, single-centre study was to investigate the clinical and laboratory features and disease outcomes of 547 patients diagnosed with primary Sjögren's syndrome (pSS) between 1975 and 2010. The patients were followed up for 11.4 ± 6.2 years. We evaluated the clinical and laboratory features, and assessed their influence on the time of diagnosis, survival, and mortality ratios, and compared them within subgroups defined by gender, glandular and extraglandular manifestations (EGMs), associated diseases, and immunoserological abnormalities. The most frequent EGMs were polyarthritis, Raynaud's phenomenon, and vasculitis among our patients; the most common associated disease was thyroiditis. During the follow-up period, 51 patients died; the median survival time was 33.71 years. Our results revealed a negative effect of cryoglobulinemia on survival ratios; additionally, the presence of vasculitis and lymphoproliferative diseases at the time of diagnosis increased the risk of mortality. The development of vasculitis was the most powerful predictor of mortality. Mortality in the group of patients with extraglandular symptoms was two- to threefold higher than in the glandular group. Attention is drawn to the importance of close monitoring and targeted diagnostic approaches in those pSS subgroups with obviously increased mortality risk.

## 1. Introduction

With a prevalence of 0.2%–1.4%, primary Sjögren's syndrome (pSS) is one of the most common chronic, slowly progressing systemic autoimmune diseases; its main symptoms are keratoconjunctivitis sicca and xerostomia. The extremely various clinical picture is characterized by multiple extraglandular manifestations (EGMs) and associated diseases. This disease affects predominantly middle-aged women, the gender ratio being 9-10 to 1; however, both distributions by age and by gender show geographical and ethnic differences [1–4].

Based on its prevalence and clinical variety, pSS is high on the list of systemic autoimmune diseases; however, there are only few publications on factors influencing mortality. According to the available literature data, geographical characteristics, ethnic groups, number of patients, studied aspects, and follow-up periods show significant differences. The applied classification criteria (particularly in studies performed before 2002) are not coherent in studies, making the comparison difficult. Investigations focusing on defining risk factors predisposing to development of lymphoproliferative diseases (LPD) are reported in several studies [5–11]; therefore, we cannot review all of them in detail.

A long-term follow-up study called attention to frequent concomitant occurrence of LPD, glomerulonephritis, and hypocomplementemia, as well as cutaneous vasculitis and mixed cryoglobulinemia. The mortality was twofold higher compared to normal population, and hypocomplementemia, mixed cryoglobulinemia, and purpuras were considered as adverse prognostic factors [12].

Davidson et al. emphasized the high incidence of thyroiditis with hypothyreosis in patients with seronegative pSS and the role of certain serological abnormalities (antinuclear antibody (ANA) or rheumatoid factor (RF) positivity) in increased tendency to progression toward rheumatoid arthritis (RA) or systemic lupus erythematosus (SLE). They have confirmed that the risk for LPD is increased in pSS patients with parotidomegaly, lymphadenomegaly, and antibody positivity to Sjögren's syndrome-specific nuclear and cytosolic antigens (anti-SS-A/-SS-B) [13].

In a large population of pSS patients, after solid tumours, the second most common cause of death was LPD, which manifested before the diagnosis of pSS in 40% of cases. An unfavourable histological finding was considered to be the most powerful predictor of mortality, and other listed factors included lymphadenopathy, palpable purpura, parotidomegaly, low complement 4 (C4) levels, and positivity for RF and ANA. The establishment of a “high-risk” or “type I autoimmune epithelitis” risk group was recommended in patients with parotidomegaly, palpable purpura, seropositivity for anti-SS-A or anti-SS-B, or hypocomplementemia, who are at high risk of developing an LPD. Additionally, it has been noted that LPD tends to develop concomitantly with glomerulonephritis and peripheral neuropathy [14].

Studies described differences also in the geographic variation of disease. An investigation in Asia revealed that unlike other geographical areas, the onset of pSS occurs earlier (at 30–39 years of age) in China; the percentage of patients with systemic symptoms is significant, above 90%. The incidence of lung and kidney involvement, pericarditis, myositis, and pancreatitis is high, while the chance for developing Raynaud's phenomenon is minimal. Thyroiditis was recorded in one third of patients. Infections, especially pneumonia, accounted for more than 60 percent of deaths. Mortality caused by liver insufficiency was manyfold higher than that of cardiovascular or cerebrovascular origin. Pulmonary artery hypertension (PAH), interstitial lung disease, liver cirrhosis, and consistently high immunoglobulin M (IgM) levels were indicated as mortality risk factors [15].

The purpose of our retrospective study was to assess the clinical and laboratory features and the disease outcomes of 547 pSS patients. We evaluated the influence of the main disease features on the time of diagnosis and mortality ratios.

## 2. Patients and Methods

### 2.1. Patients

The Division of Clinical Immunology of the University of Debrecen is one of the largest tertiary referral centers in Hungary for systemic autoimmune diseases. In the present study, we collected all the patients who were diagnosed and followed up regularly with primary Sjögren's syndrome between 1975 and 2010 at our center. From the whole group of these 1094 patients, we gained a random sample by using a systemic sampling method. After arranging their name in alphabetical order, we selected every second patient for the analyses. Consequently, the final population of our retrospective study consisted of 547 (487 women and 60 men, gender ratio = 8 to 1) patients with pSS. Before 2002, Fox RI criteria [16, 17] were used for establishing the diagnosis; after 2002, American-European consensus criteria (AECC) were used [18]. Cases diagnosed before 2002 were revised according to AECC. Patients were followed up every 6 months at our outpatient clinic for autoimmune diseases. All experiments carried out in the study were in compliance with the declaration of Helsinki.

### 2.2. Clinical Evaluations and Laboratory Measurements

During follow-up visits, EGMs, associated diseases, and immunoserological characteristics were recorded. Immunoserological tests were performed at the Regional Immunology Laboratory of the University of Debrecen and included the measurement of ANA, RF, antibodies against extractable nuclear antigen (ENA), anti-SS-A, anti-SS-B, anti-DNA, anti-thyroglobulin (TG), anti-thyroid peroxidase (TPO) antibody, serum immunoglobulin, cryoglobulin, and complement levels. The quantitative measurement of autoantibodies (anti-ENA, anti-SS-A/-SS-B, anti-CCP, anti-TG, anti-TPO, ANA, RF, and anti-DNA) was performed with enzyme-linked immunosorbent assay (ELISA) technique. Immunoglobulin levels and complement activity were determined with turbidimetry and nephelometry techniques and haemolysis test in sheep red blood cell suspension, respectively. The presence of each element and its changes in time were monitored in the whole study population. Data were evaluated by comparing well-defined subgroups (women/men, glandular/EGM, presence/absence of associated diseases, presence/absence of immunoserological differences, and patients alive at the end of the study/deceased over time).

### 2.3. Statistical Analyses

The SPSS version 20.0 (SPSS Inc., Chicago, IL, UDA) was used for statistical analysis. To analyze the distribution of the data, Kolmogorov-Smirnov test was used. In cases of normal distribution, we determined mean ± standard deviation (SD) values and used two-sample *t*-test for statistical evaluation of the experimental data. In cases of nonnormal distribution, median, minimum, and maximum values were calculated, and Mann-Whitney *U* test was used.

Survival time and rate were assessed using Kaplan-Meier estimator. Chi-square test and Fisher's exact test were used to discriminate between patient groups; we used Cox regression model to predict poor outcome of the disease. For comparison among patient and control groups, standardized mortality ratios (SMRs) were calculated. Differences were considered statistically significant at *P* < 0.05.

## 3. Results

### 3.1. Patients' Characteristics

The demographic characteristics of patients' groups are demonstrated in Tables 1 and 2. The mean follow-up period was 11.4 ± 6.2 years with a range from 2 to 37 years. There was no significant difference in the mean age at the time of pSS diagnosis and in the follow-up period between males and females. The mean age at the time of diagnosis of the 51 deceased patients was significantly higher than for the 496 patients still alive at the end of the study, while there were no significant differences in follow-up periods between the two groups. Interestingly, we statistically confirmed that when at least one EGM was present, pSS was diagnosed 3.5 years earlier on average than in the glandular subgroup (Table 1). We did not observe significant difference between the sex ratios and the presence of EGMs in patient groups stratified by age (Table 2).

### 3.2. Clinical and Laboratory Features

**Figure 1:**
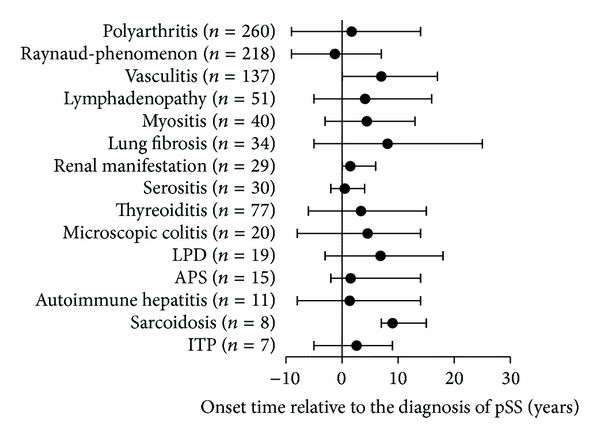
Time intervals between the diagnosis of the primary Sjögren's syndrome and the onset of extraglandular manifestations and associated diseases.

Table 3 summarizes the clinical manifestations and immunoserological findings of pSS patients. The three leading EGMs each affecting more than one quarter of patients were polyarthritis, Raynaud's phenomenon, and vasculitis. The frequency of lymphadenopathy, myositis, pulmonary fibrosis, renal manifestations, and serositis ranged between 5.3% and 9.3%. The onset of certain EGMs (e.g., polyarthritis, Raynaud's phenomenon, and pulmonary fibrosis) might precede the establishment of pSS diagnosis even with 5–9 years (Figure 1). Raynaud's phenomenon and serositis were manifested typically in the early phases of the autoimmune disease; a great proportion of cases were already present when the diagnosis of pSS is established. Vasculitis and renal manifestations usually developed after the diagnosis of pSS. With an incidence of 13.9%, thyroiditis was the most common associated disease. The occurrence of other associations (microscopic colitis, LPDs, APS, autoimmune liver diseases, sarcoidosis, and ITP) was lower than 4% in our study (Table 3). APS and autoimmune liver diseases often preceded the onset of pSS, while sarcoidosis was characterized by late manifestation (Figure 1). The pSS predominantly occurred in perimenopause; additionally, in most cases, the investigated EGMs and associated diseases also developed in this time interval. As an exception, in some cases (lymphadenopathy, lung fibrosis, renal manifestations, microscopic colitis, thyroiditis, and LPDs), incidence peaked between 40 and 49 years of age.

Approximately, 55%–75% of pSS patients were positive for ENA, anti-SS-A, ANA, and anti-SS-B or had hypergammaglobulinemia. RF positivity and hypocomplementemia occurred in more than 20% of cases, while more than 10% of patients were anti-TPO and anti-DNA positive. Other tested parameters (anti-TG, anti-CCP, and cryoglobulin) were positive in 6–9 percent of cases (Table 3). Significant gender differences were found for eight investigated factors, six of which were predominant in women (Raynaud's phenomenon, thyroiditis, anti-SS-B, anti-DNA, anti-TG, and anti-TPO) and two in men (polyarthritis, RF) (Table 3).

### 3.3. Disease Outcomes

**Figure 2:**
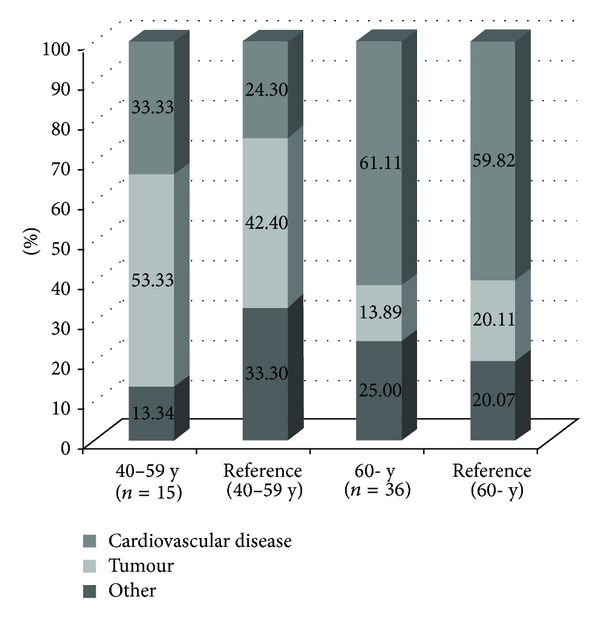
Comparison of the causes of death between our patients' data and the sex- and age-adjusted data on general Hungarian population.

During the follow-up period, 51 (46 women and 5 men) of our patients died. Mortality of the whole patient population was almost 9 percent, with no significant differences between genders. As to the distribution by age groups, we lost 36 (32 female and 4 male) and 15 (14 female and 1 male) patients, from the >60 year and 40–59 year age groups, respectively. When evaluating the causes of death, cardiovascular events (myocardial infarction, pulmonary embolism, and stroke) were the leading causes, being followed by solid tumours (bronchial, colorectal, and bladder carcinoma, as well as invasive ductal breast cancer and malignant melanoma). Various causes (infection, ileus, gastrointestinal bleeding, and suicide) also accounted for some deaths.  Figure 2 demonstrates the comparison of our patients' data and the sex- and age-adjusted data of the general Hungarian population, based on the report of the Hungarian Central Statistical Office from 2007 [20]. According to our observations, SS hardly affected the trend of cause of death in our patient population.

Calculated mortality per 1,000 individuals per year was as follows: in the Hungarian population adjusted for age and gender ratios of the whole pSS population 7.821 (based on the data of the Hungarian Central Statistical Office from 2001 [19]); in the whole pSS population 10.360; among female patients 10.495; among male patients 9.259. Additionally, calculated mortality per 1,000 individuals in the EGM subgroup (11.887) was two-and-a-half-fold higher than that in the glandular group (4.752). Standardized mortality ratios (SMRs) were also assessed in the whole pSS population, and separately for women and men, and for glandular and EGM subgroups as well (based on the data of the Hungarian Central Statistical Office from 2001 [19]) (Table 4).

**Figure 3:**
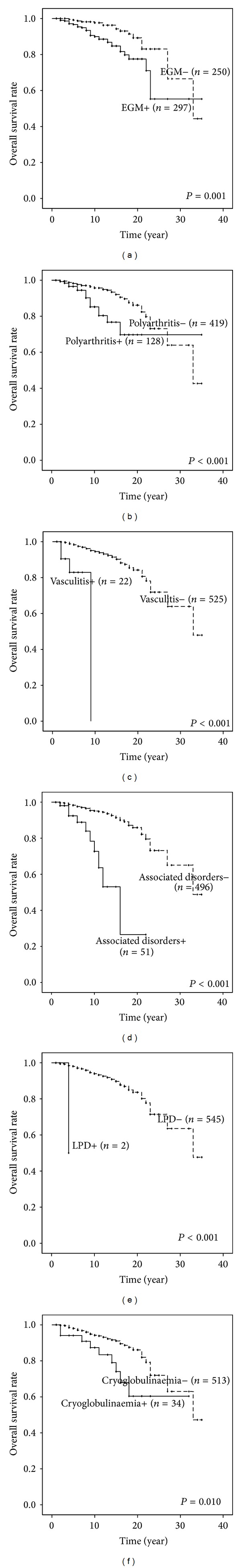
The Kaplan-Meier survival plots for the risk of death in patients subgroups with/without (a) extraglandular manifestations, (b) polyarthritis, (c) vasculitis, (d) associated disorders and (e) lymphoproliferative disease developed already at the time of diagnosis, or (f) cryoglobulinemia developed during the disease course.

Median survival time in the whole population was 33.71 years. Patients with pSS, complicated from the time of diagnosis with EGM or associated diseases, could be characterized with significantly worse survival ratios. This was also valid in the case of early occurrence of polyarthritis, vasculitis, and LPD. Late occurrence of cryoglobulinemia (even years after the diagnosis of pSS) also impaired survival ratios significantly (Figures 3(a)–3(f)). In the above listed cases, estimated median survival time is obviously shortened, compared to patients with uncomplicated pSS, at the higher extent in the presence of LPD and vasculitis (Table 5).

Mortality risk in subgroups with significantly worse survival ratios increased 1.085–10.716-fold. An older age at the time of pSS diagnosis also increased the risk for death, numerically by 8.5 percent per year. The presence of vasculitis before the diagnosis of pSS resulted in the highest risk, while the lowest risk was associated with a younger age at onset of pSS (Table 6).

## 4. Discussion

In our retrospective study, we reported on research outcomes of 547 patients with pSS from one Hungarian clinical immunology centre. No other research on pSS patients with similar ethnical characteristics has been conducted in any East-Central European centre before. Compared to earlier publications, our pSS population was the third largest, but it was the first, when considering the patient proportion related to the whole population of the given countries [12, 14, 15]. The spectrum of our study (the number of factors potentially influencing the clinical outcome and mortality) was broad, investigating more aspects than prior studies. The value of our observations is increased by the long follow-up period.

In Greek publications, the proportion of female patients was high (women to men ratio = 16–22 : 1); in British and Chinese studies it follows internationally accepted rates, while, in Hungary, the proportion of men is higher than usual (women to men ratio = 8 : 1). The mean age of our patients at the time of pSS diagnosis is in line with the British values. In Southern Europe, the disease sets on 2–5 years later, while in China 8–10 years earlier [12–15]. The influence of the gender, the presence of EGMs and associated diseases on the time of pSS diagnosis, and the typical time intervals for the manifestation of clinical features were investigated only by our working group, leading to new findings. The diagnosis of pSS is established circa 2.5 years earlier in men, while in the presence of at least one EGM, it is made 3.5 years earlier, irrespective of the gender. In accordance with this finding, it is also noted that, in women, pSS begins more often with tolerable sicca syndromes, explaining the delayed seek for help, while in men, a severe EGM may occur as the first symptom.

In our study, we evaluated which EGMs and associated diseases, in which age groups, at what typical time intervals, and in what proportion occurred among our patients. The three leading EGMs were polyarthritis, Raynaud's phenomenon, and vasculitis. The frequency of other EGMs ranged between 5% and 10%. When comparing our results to the literature data, we can conclude that, in the Hungarian population, the order of frequency for EGM is similar to what is seen in British people; the frequency of polyarthritis and vasculitis correlates with Chinese data, while that of Raynaud's phenomenon and pulmonary fibrosis corresponds to Greek data. Lymphadenopathy and renal manifestations occurred in a lower proportion among our patients; the incidence of myositis was higher than in the literature, while serositis developed in an approximately similar proportion. With few exceptions, EGMs tend to precede the onset of systemic autoimmune disease, as if anticipating it. The newly defined characteristic manifestation time intervals of each EGM draw attention to the importance of cooperation with related professions in conditions predicting pSS. Significant gender differences were found for two EGMs: polyarthritis with the predominance in males and Raynaud's phenomenon predominating in females. The presence of EGMs enabled an earlier establishment of the diagnosis.

During many decades of care activity, we concluded that certain associated diseases worsen the course of pSS; therefore, we decided to analyse them with the method applied for EGM. The most common associated disease in our patients was thyroiditis, while incidence of other associated diseases was below 3%-4% [21]. When comparing the frequency of associated disease with the few available literature data, the following can be concluded. The percent incidence of thyroiditis in the Hungarian and British population [13] was approximately the same, and in Chinese people [15] it was more than twofold as compared to Hungarians, while in the Greek population no such investigation was performed. The incidence of LPD in Hungarian pSS patients was in line with Greek values, in British people it was 1 percent, and it was not measured for the Chinese population. Autoimmune liver diseases occurred half as frequently in our patients, when compared to Greek, British, and Chinese data. The risk group and the screening tests for autoimmune liver diseases have been defined by our working group in pSS previously [22]. Apart from our working group, APS was evaluated only by Chinese researchers [15], who found a two- to threefold higher prevalence compared to the Hungarian population. Our data regarding association with microscopic colitis, sarcoidosis, and ITP appear for the first time in the literature. The chronic low-grade inflammation typical for pSS might cause the remodeling of gut wall which explains the association with microscopic colitis, as our working group published in 2005 [23].

Differences in the prevalence of EGMs and associated diseases modifying the clinical picture, as compared to literature data, may be explained by the influence of different genetic, life style, and geographical factors. In 2002, we reviewed non-Hodgkin's lymphoma (NHL) patients to describe its coexistence with autoimmune diseases. The most frequent autoimmune disease in the NHL group was pSS. This association can be explained by the dysregulation of apoptosis and increased levels of activated B-cells [24]. Our results regarding the distribution of associated diseases by gender and age group and their influence on survival and mortality ratios are new, and no similar analysis has been performed earlier.

Significant gender differences were found for several serological factors. Anti-TG and anti-TPO positivity prevailed in females, while RF showed male predominance, in accordance with the observed clinical differences, and with the dominance of thyroiditis in women and polyarthritis in men. Cryoglobulinemia can be considered a highly relevant immunoserological abnormality, the emergence of which in the follow-up period of pSS significantly impairs survival ratios and increases mortality risk. In our previous reports, we concluded that cryoglobulinemia may play a more important role in the extraglandular features observed in SS associated with hepatitis C virus (HCV) than it does in pSS alone, although these manifestations also might be related to either the underlying SS or the HCV infection itself [25, 26]. Interestingly, the mortality of the whole patient population was comparable to that of normal Hungarian population, demonstrating the adequacy of care and a relatively favourable course of the disease.

## 5. Conclusion

Summarizing our results, we concluded that pSS is composed of subgroups displaying a different clinical picture and mortality risk. During our work, we identified clinical and immunoserological features characterizing Hungarian patients. Based on significantly worse survival ratios and the concomitantly increasing mortality risk, pSS subgroups with polyarthritis, vasculitis, LPD, or cryoglobulinemia should be clinically classified as severe pSS. Consequently, we recommend the use of targeted diagnostic protocols for identifying patients with severe pSS. Moreover, close observation of cases associated with polyarthritis, vasculitis, LPDs, or cryoglobulinemia is also essential.

## Figures and Tables

**Table 1:** The mean age of patients determined at the time of diagnosis and the average follow-up period.

Subgroup	Mean age at the time of diagnosis of pSS (years)	Follow-up period (years)
Male (*n* = 60)	47.55 ± 12.051	11.47 ± 6.113
Female (*n* = 487)	49.99 ± 11.366	11.41 ± 6.342
*P* value	**NS**	**NS**
Alive (*n* = 496)	49.14 ± 11.249	11.41 ± 6.240
Deceased (*n* = 51; male: 5; female: 46)	55.35 ± 12.038	11.49 ± 7.035
*P* value	**<0.001**	**NS**
EGM (*n* = 430)	48.97 ± 11.273	12.07 ± 6.408
Glandular (*n* = 117)	52.48 ± 11.751	9.04 ± 5.331
*P* value	**0.003**	**<0.001**

*P* values indicate differences between genders, alive and deceased patients, and also extraglandular and glandular subgroups.

**Table 2:** Percentages of sex and clinical characteristics in patient groups stratified by age.

Age at diagnosis years	Patients' number	Female		Male		Glandular		EGM	
		Number	%	Number	%	Number	%	Number	%
0–29	27	23	85,19	4	14,81	6	22,22	21	77,78
30–39	70	58	82,86	12	17,14	10	14,29	60	85,71
40–49	154	136	88,31	18	11,69	26	16,88	128	83,12
50–59	183	169	92,35	14	7,65	39	21,31	144	78,69
60-	113	101	89,38	12	10,62	36	31,86	77	68,14

**Table 3:** Frequency of clinical and immunoserological features during the disease course.

Clinical and serological features	Frequency (%)	Distribution according to gender (*n*)		*P* value
		Female (*n* = 487)	Male (*n* = 60)	
EGMs				
Polyarthritis (*n* = 260)	48.1	218	42	<0.001
Raynaud's phenomenon (*n* = 218)	39.9	213	5	<0.001
Vasculitis (*n* = 137)	25	126	11	0.203
Lymphadenopathy (*n* = 51)	9.3	46	6	0.89
Myositis (*n* = 40)	6.9	35	5	0.748
Lung fibrosis (*n* = 34)	6.2	31	3	0.679
Renal manifestation (*n* = 29)	5.5	26	3	0.912
Serositis (*n* = 30)	5.3	29	1	0.169
Associated disorders				
Thyroiditis (*n* = 77)	13.9	77	0	0.001
Microscopic colitis (*n* = 20)	3.5	17	3	0.436
LPD (*n* = 19)	3.3	15	4	0.152
APS (*n* = 15)	2.7	15	0	0.168
Autoimmune hepatitis (*n* = 11)	1.6	10	1	0.840
Sarcoidosis (*n* = 8)	1.5	6	2	0.201
ITP (*n* = 7)	1.3	7	0	0.350
Serological positivity				
ANA (*n* = 353)	64.6	316	37	0.623
anti-ENA (*n* = 432)	78.9	382	50	0.38
anti-SS-A (*n* = 421)	76.9	372	49	0.359
anti-SS-B (*n* = 302)	55.2	277	25	0.025
anti-DNA (*n* = 75)	13.7	72	3	0.038
RF (*n* = 163)	29.8	130	33	<0.001
anti-CCP (*n* = 40)	7.3	34	6	0.397
anti-TG (*n* = 48)	8.8	48	0	0.011
anti-TPO (*n* = 102)	18.5	101	1	<0.001
Hypergammaglobulinemia (*n* = 381)	69.6	338	43	0.719
Hypocomplementemia (*n* = 119)	21.8	106	13	0.986
Cryoglobulinemia (*n* = 34)	6.2	32	2	0.567

*P* values indicate differences between genders. LPD: lymphoproliferative disorders; APS: antiphospholipid syndrome; ITP: immune thrombocytopenic purpura; ANA: antinuclear antibody, ENA: antibody to extractable nuclear antigen; DNA: anti-DNA antibody; RF: rheumatoid factor; TG: antibodies to thyroglobulin; TPO: antibodies to thyroid peroxidase.

**Table 4:** Standardized mortality ratios (SMRs) of pSS subgroups.

	Standardized mortality ratios (SMRs)
Whole pSS population (*n* = 547)	1.32
Female patients (*n* = 487)	1.49
Male patients (*n* = 60)	0.65
Patients without EGMs (*n* = 117)	0.51
Patients with EGMs (*n* = 430)	1.62

Calculations were based on the data of the Hungarian Central Statistical Office from 2001 [19].

**Table 5:** Variations in survival times for parameters that are significantly impairing survival indicators compared to the average values of patients.

	*P* value, when a certain factor is present at the time of pSS diagnosis	*P* value, when a certain factor manifests after the diagnosis of the pSS	Survival time (year)	CI 95%
Mean survival time for our patient with pSS			33.71	
Studied factor				
EGM	0.001	NS	26.949	23.907–29.991
Polyarthritis	<0.001	NS	27.554	23.844–31.263
Vasculitis	<0.001	NS	7.956	6.700–9.213
Associated disorder	<0.001	NS	14.283	10.962–17.603
LPD	<0.001	NS	4.000	4.000-4.000
Cryoglobulinemia	NS	0.010	24.112	19.899–28.326

CI: confidence interval; NS: nonsignificant.

**Table 6:** Mortality risk in subgroups with significantly worse survival indicators, also taking into consideration the age recorded at the diagnosis.

Analysed variable	*P* value	Relative risk	CI 95%
Age at the diagnosis of pSS (continuous variable)	<0.001	1.085*	1.049–1.121
Polyarthritis	0.048	1.898	1.006–3.581
Vasculitis	0.001	10.716	2.795–41.089
LPD	0.005	5.172	1.652–16.192
Cryoglobulinemia	0.038	2.331	1.048–5.185

*With one year increase in age the risk for death is 1.085-fold higher.
